# ARTIS Pheno®: a potential tool for cochlear implant surgery

**DOI:** 10.1007/s00405-024-08588-y

**Published:** 2024-04-12

**Authors:** S. Schmidt, M. Tisch, K. Bahr-Hamm, C. Matthias, D. Overhoff, S. Waldeck

**Affiliations:** 1 Department of Otolaryngology, Bundeswehr Central Hospital, Koblenz, Germany; 2 Department of Otolaryngology, Bundeswehr Hospital, Ulm, Germany; 3 Department of Otolaryngology, Johannes Gutenberg University Medical Centre, Mainz, Germany; 4 Department of Radiology and Neuroradiology, Bundeswehr Central Hospital, Koblenz, Germany

**Keywords:** Petrous bone abnormalities, High quality of care, Quality control, ARTIS Pheno, Cochlear implantation

## Abstract

**Purpose:**

Cochlear implantation is a standard approach to hearing rehabilitation and encompasses three main stages: appropriate patient selection, a challenging surgical procedure, which should be as atraumatic as possible and preserve cochlear structures, and lifelong postoperative follow-up. Computed tomography (CT) is performed to assess postoperative implant position. The Siemens Advanced Radar Target Identification System (ARTIS) Pheno provides fluoroscopic imaging during surgery and has so far been mainly used by cardiologists, neurosurgeons and trauma surgeons.

**Methods:**

Six patients with difficult anatomy or a challenging medical history were selected for a surgical procedure, during which we planned to use the ARTIS Pheno to accurately position and assess implant position under fluoroscopy during and immediately after surgery. In all six cases, the ARTIS Pheno was used directly in the surgical setting. The procedures were performed in cooperation with the neuroradiology department in an interdisciplinary manner.

**Results:**

In all six patients, fluoroscopy was used to visualise the procedure at different stages of surgery. In five patients, the procedure was successfully completed. This approach allowed us to finally assess implant position and confirm the correct and complete insertion of the electrode while the patient was still under anaesthesia.

**Conclusion:**

These cases showed positive surgical outcomes. Although the procedure is more complex than a standard approach, patients can be managed in a safe, effective and appropriate manner. The assessment of implant position in real time during surgery leads to greater patient and surgeon satisfaction. The approach presented here ensures a high quality of cochlear implant surgery even in difficult surgical situations and meets the requirements of modern surgery.

## Introduction

Increasing professionalism in cochlear implantation should include the standardisation and professionalisation of cochlear implant surgery under particularly challenging conditions. The Siemens Advanced radar Target Identification System (ARTIS) Pheno, together with other useful and/or necessary instruments and support systems from various medical technology suppliers (e.g. surgical microscopes, endoscopes, neuromonitoring, drilling and navigation systems), can help to achieve this goal [5]. Surgical teams have a wide range of technical options at their disposal (state-of-the-art anaesthesiatic equipment, etc.).

Some of our patients have a history of multiple previous surgeries that have altered to important anatomical structures. Our aim is to understand and respect these anatomical changes and to improve the patient’s situation without causing additional damage. In particular, of the increasing use of technology and robotic surgery in cochlear implantation will reduce, the number of specialists in this field. In this situation, imaging modalities can provide helpful support.

We present the example of cochlear implantation to demonstrate how technical innovations are applied by an interdisciplinary team in an integrated operating room equipped with state-of-the-art devices and interventional radiology equipment. We have attempted to apply theoretical ideas in a practical settings and to assess the potential strengths and weaknesses of our approach at different stages of surgery. Cochlear implantation is well suited for this purpose because it is a surgically and anatomically challenging yet clearly structured procedure that involves the use of a wide variety of technical tools.

## Materials and methods

**Fig. 1:**
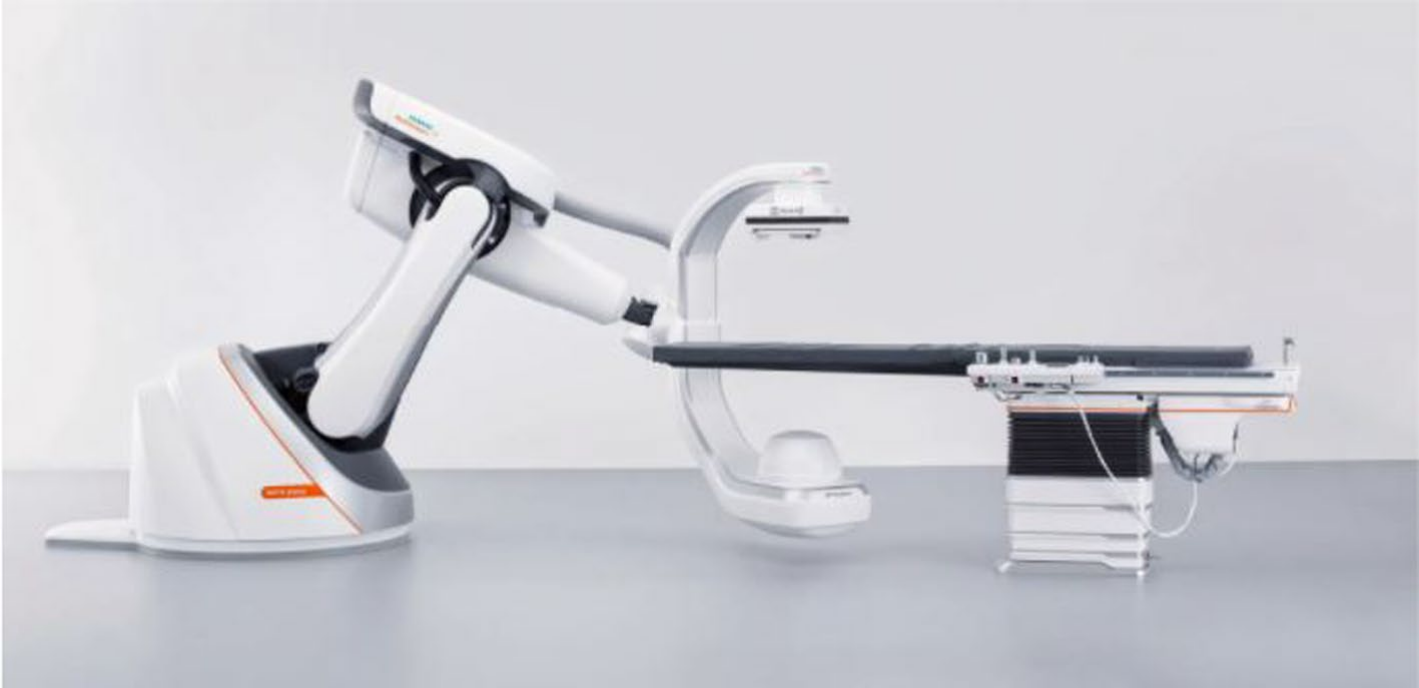
ARTIS Pheno. Source: SIEMENS Healthineers

From April 2018 to February 2019, six patients (see below) presented to our department for unilateral (*n* = 5) or bilateral (*n* = 1) hearing rehabilitation. A non-standard surgical approach was planned in the preoperative setting for patients with abnormal anatomy. Surgery was performed in the interventional suite of the radiology department, which is equipped with an ARTIS Pheno (Fig. 1) computed tomography (CT) system (Siemens Healthineers, Erlangen, Germany). The aim of this approach was to manage patients with considerably altered anatomy surgically and to use fluoroscopy to visualise the surgical procedure and to assess the implant position during and immediately at the end of the procedure. ARTIS Pheno has been used primarily in interventional radiology, cardiology, neurosurgery and trauma surgery. The system features a 100-kW generator, an a-Si detector, and a pixel size of 160 μm. It is specifically designed for personalised pre-treatment planning and decision support during treatment, with the advantage of instant quality control.

### Participants

Patient 1 experienced a sudden hearing loss with total deafness in his left ear. His hearing did not recover. Following diagnostic tests, the patient was treated conservatively with initial corticosteroid therapy. He returned to the clinic after three weeks of rehabilitation. During his stay at a cochlear implant rehabilitation centre, the patient’s hearing did not improve. As the patient’s quality of life was severely compromised by his unilateral deafness, the decision was made to implant a cochlear implant. Further imaging was not considered necessary because of the radiation exposure and the short period of time that had elapsed. In retrospect, this decision was wrong. Marked sclerotic changes were found during surgery, making it impossible to insert an electrode. In this situation, the decision was made to perform the surgery under intraoperative fluoroscopy in the Neuroradiology Department. While the patient was still under anaesthesia, the planned position, insertion angle and depth of the implant were assessed and found to be correct. However, even with interventional radiology instruments and catheters, the implant could not be placed.

Although this procedure was unsuccessful, it provided reassurance that cochlear implantation was not an option for this patient.

In a second procedure, the patient was treated with a Bonebridge^®^ implant system.

This case marked the beginning of fluoroscopy-guided ear surgery in our department.

**Fig. 2:**
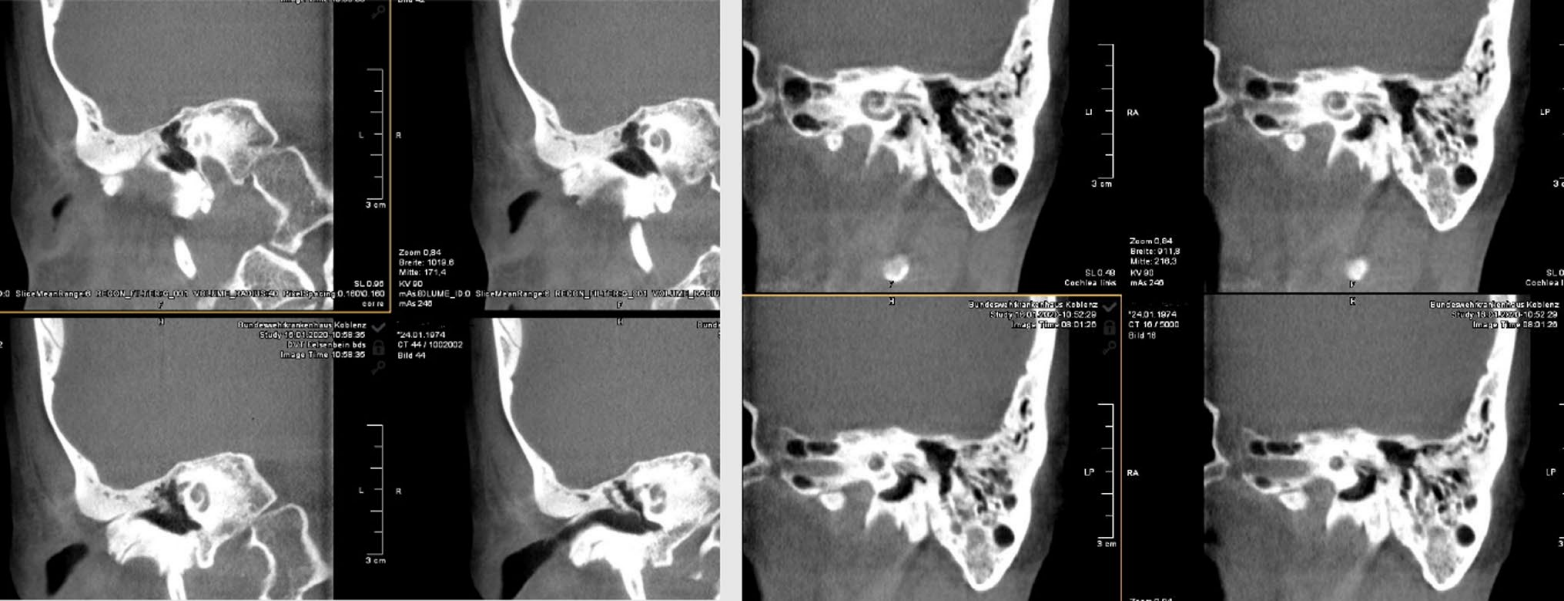
CBCT of the petrous bone of patient 2 (as an example of the surgical procedures in patients 1 and 2)

Patient 2 presented with sudden onset of bilateral deafness. Cone-beam computed tomography (CBCT) of the petrous bone (Fig. 2) showed opaque changes in both cochleae similar to those seen in patient 1. Based on these findings, it was decided to proceed with to perform surgery in the Neuroradiology Department two days later. The Implants were successfully implanted in both ears initial gelatinous changes in the round window membranes.

**Fig. 3:**
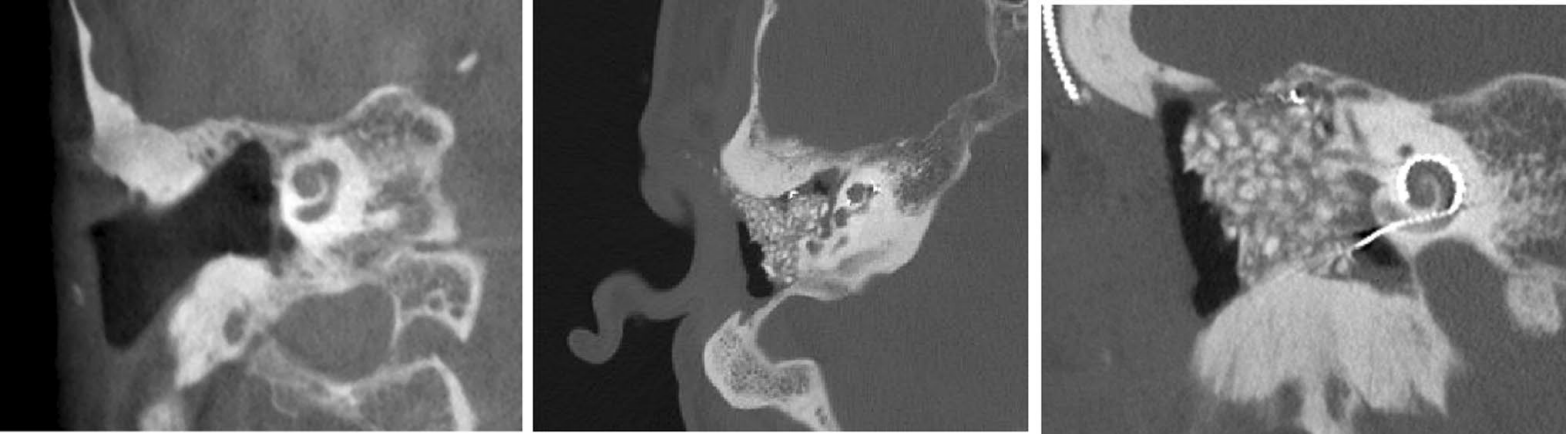
Coronal CBCT of the petrous bone of patient 4 (as an example of the surgical procedures in patients 3 and 4), **a** before surgery, **b** after surgery

In patients 3 and 4, the decision to perform surgery in the interventional suite of the neuroradiology department was made in the preoperative setting. This allowed the surgical team to obtain images during surgery (if required) and at the end of the procedure for to assess the position of the electrode prior to obliteration of the mastoid cavities, coverage of the eroded semicircular canals and exposed facial nerves, and obliteration of the auditory canals (Fig. 3). Cochlear implantation can also be performed by an experienced surgeon without imaging, but an assessment of implant position prior canal obliteration with Bonalive or abdominal fat contributes to quality assurance.

**Fig. 4:**
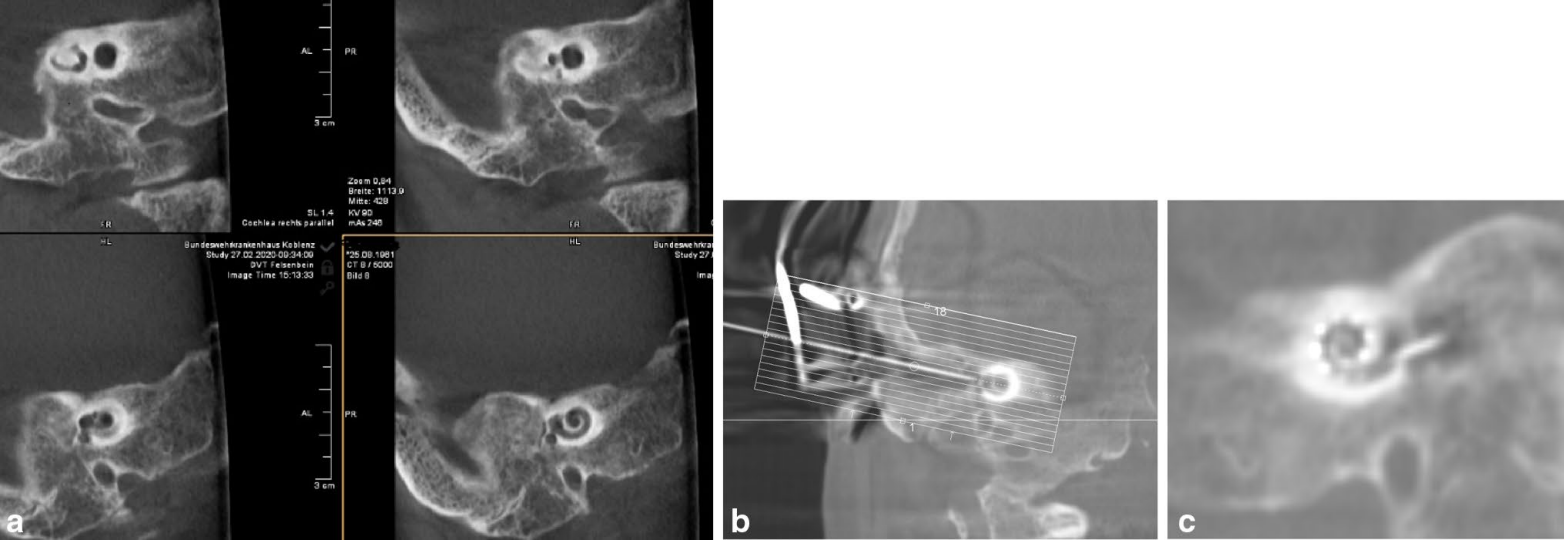
Bilateral petrous bone abnormalities in patient 5, **a** before surgery, **b** while surgery and **c** after surgery

Patients 5 and 6 presented with rare petrous bone anomalies. In both patients, the cochlea appeared normal on imaging, but it was not possible to identify the facial nerve with certainty. In addition, the anatomical landmarks of the auricle, auditory canal and middle ear structures were absent. In such a situation, it may not be possible to locate the round window membrane with certainty (Fig. 4). In addition, in patient 5, the cochlea and the vestibulocochlear nerve were absent on the contralateral side as a result of thalidomide embryopathy. The patient therefore had only one ear suitable for implant.

Therefore, in these two cases, we preoperatively planned to perform the surgery in the Neuroradiology Department.

### Equipment and procedures

Specialists from the radiology and ENT departments thoroughly discussed and determined the surgical procedure with all patients (except patient 1) all available data(CT images of the petrous bone, medical records, audiograms, surgical safety checklist, etc.).

Scheduling surgery, care was taken to ensure that the specialists who had informed the patients were present during surgery.

The preparation of the patients did not take place in the main operating theatre but in the radiology department.

Surgical equipment and supplies required for the operation (operating microscope, instrument sets, neuromonitoring and intraoperative implant assessment equipment, implant, etc.) were taken to the radiology department (Table 1).

**Table 1:** Overview of patients

Patient	Medical condition	Problem(s)	Imaging	Surgery
Pat. 1 (male, 49 years)	Sudden hearing loss in his left ear and deafness	Electrode insertion was impossible during the surgical procedure	CBCT of the petrous bone: sclerotic changes, signs of ossification	Unsuccessful cochlear implant surgery Insertion of a Bonebridge implant system in a secondary procedure
Pat. 2 (female, 36 years)	Sudden hearing loss and sudden deafness in both ears	Sudden total deafness, no recovery Virus serology: acute, highly avid cytomegalovirus (CMV) infection Mild mumps infection	CBCT of the petrous bone: sclerotic changes in both ears	Simultaneous bilateral cochlear implantation
Pat. 3 (female, 68 years)	Cholesteatomas	Eight surgical procedures for cholesteatoma, construction of a large mastoid cavity, exposed semicircular canals, functional deafness	CBCT of the petrous bone	Cochlear implantation and obliteration of the mastoid cavity with abdominal fat
Pat. 4 (male, 53 years)	Cholesteatomas	Twelve surgical procedures including the construction of a mastoid cavity Facial nerve irritation caused by thermal stimuli, facial numbness Erosion of two semicircular canals, dizziness Magnetic plate of a bone conduction hearing aid	CBCT of the petrous bone MRI of the skull following removal of the magnetic plate	Removal of a Medtronic magnetic plate Cochlear implantation Coverage of eroded semicircular canals Separation of the facial nerve from the tympanic membrane Obliteration of the mastoid cavity with Bonalive Obliteration with abdominal fat in a further procedure
Pat. 5 (male, 60 years)	Thalidomide embryopathy Anotia, auditory canal atresia	Bilateral deafness, bilateral petrous bone abnormalities Middle ear malformation, normal appearance of the cochlea, progressive inner ear hearing loss Insufficient benefit from the use of a bone conduction hearing aid	MRI of the skull CBCT of the petrous bone	Cochlear implantation (right ear)
Pat. 6 (male, 74 years)	Congenital auditory canal atresia, anotia Creation of an auricle and auditory canal in six procedures during childhood	Middle ear malformation Poorly identifiable but functionally intact facial nerve Normal appearance of the cochlea	MRI of the skull CBCT of the petrous bone	Cochlear implantation (right ear)

Following standard surgical site disinfection and in collaboration with the radiologists, the patient was wrapped in sterile drapes to prevent contamination of the sterile field when the ARTIS Pheno C-arm was rotated during fluoroscopy.

The operating microscope, and the and the neuromonitoring and anaesthesia equipment were positioned in such a way that they did not obstruct the C-arm.

During fluoroscopy, all medical staff left the interventional suite. They observed the patient and monitored all vital parameters through a large window from an adjacent room. A radiologist and a radiology technologist, both wearing protecting clothing, operated the entire operating table, including the C-arm fluoroscope.

Fluoroscopy was generally performed after posterior myringotomy and after insertion of the cochlear implant electrode and, if necessary, to assess the insertion angle and the position in the mastoid.

**Fig. 5:**
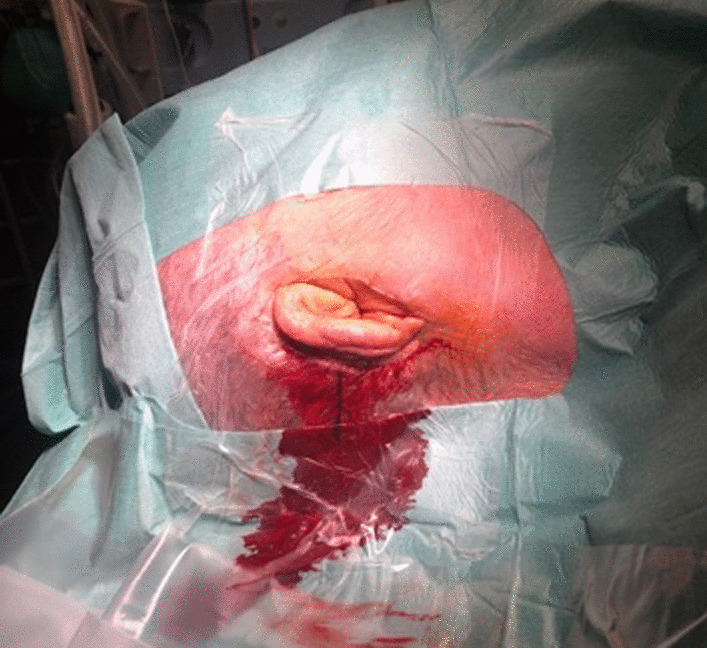
Photo of the reconstructed auricle

**Fig. 6:**
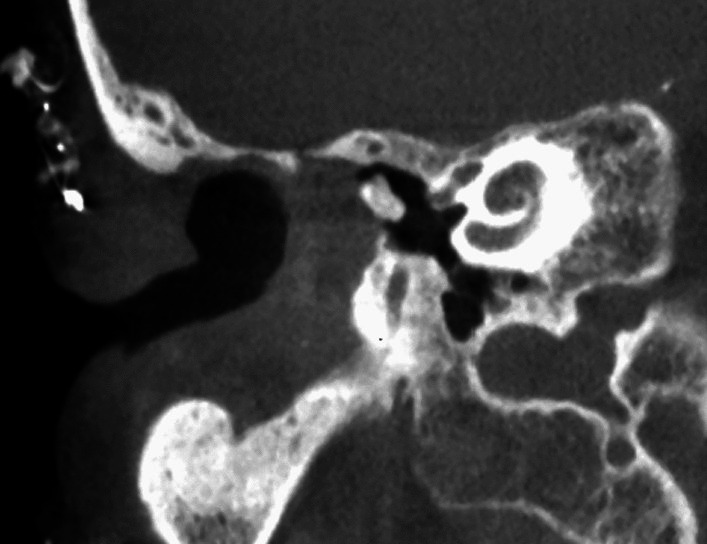
Preoperative coronal CBCT of the petrous

**Fig. 7:**
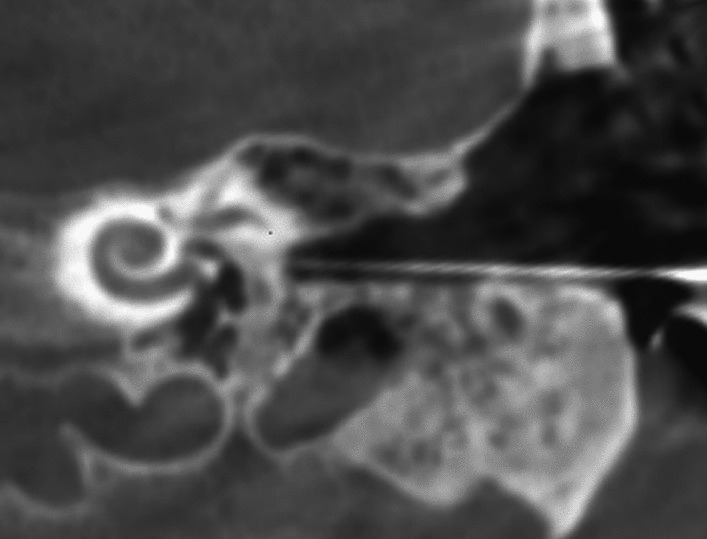
Intraoperative fluoroscopy using a thin bone needle

**Fig. 8:**
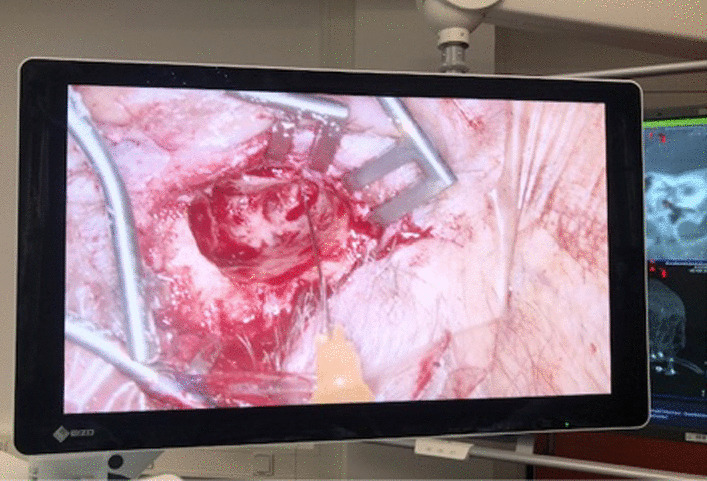
Intraop photo showing needle advancement

**Fig. 9:**
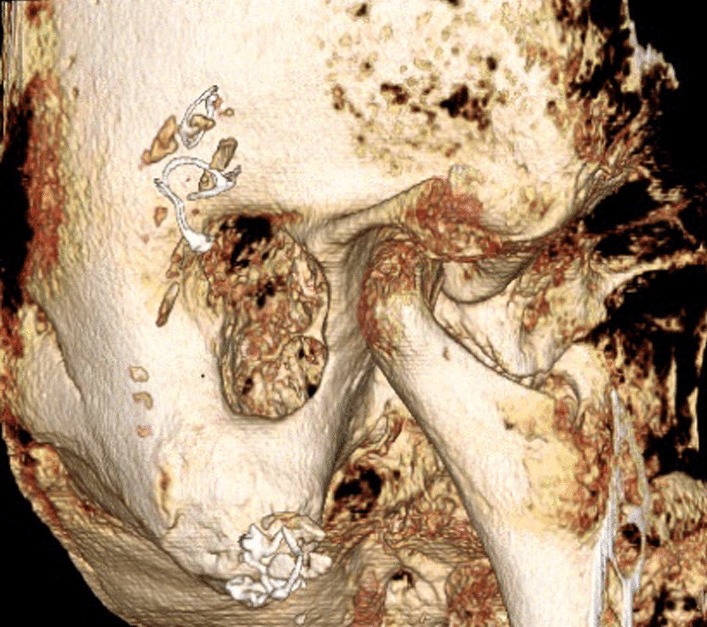
3D- image of the petrous bone

**Fig. 10:**
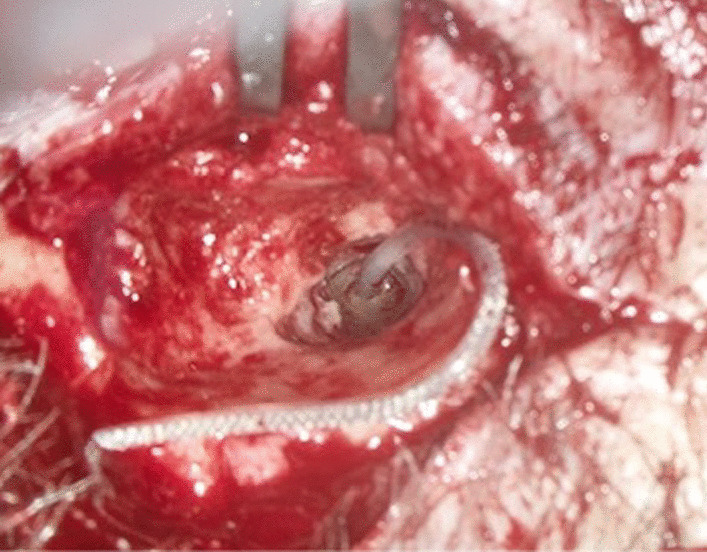
Intraoperative photo with the electrode

**Fig. 11:**
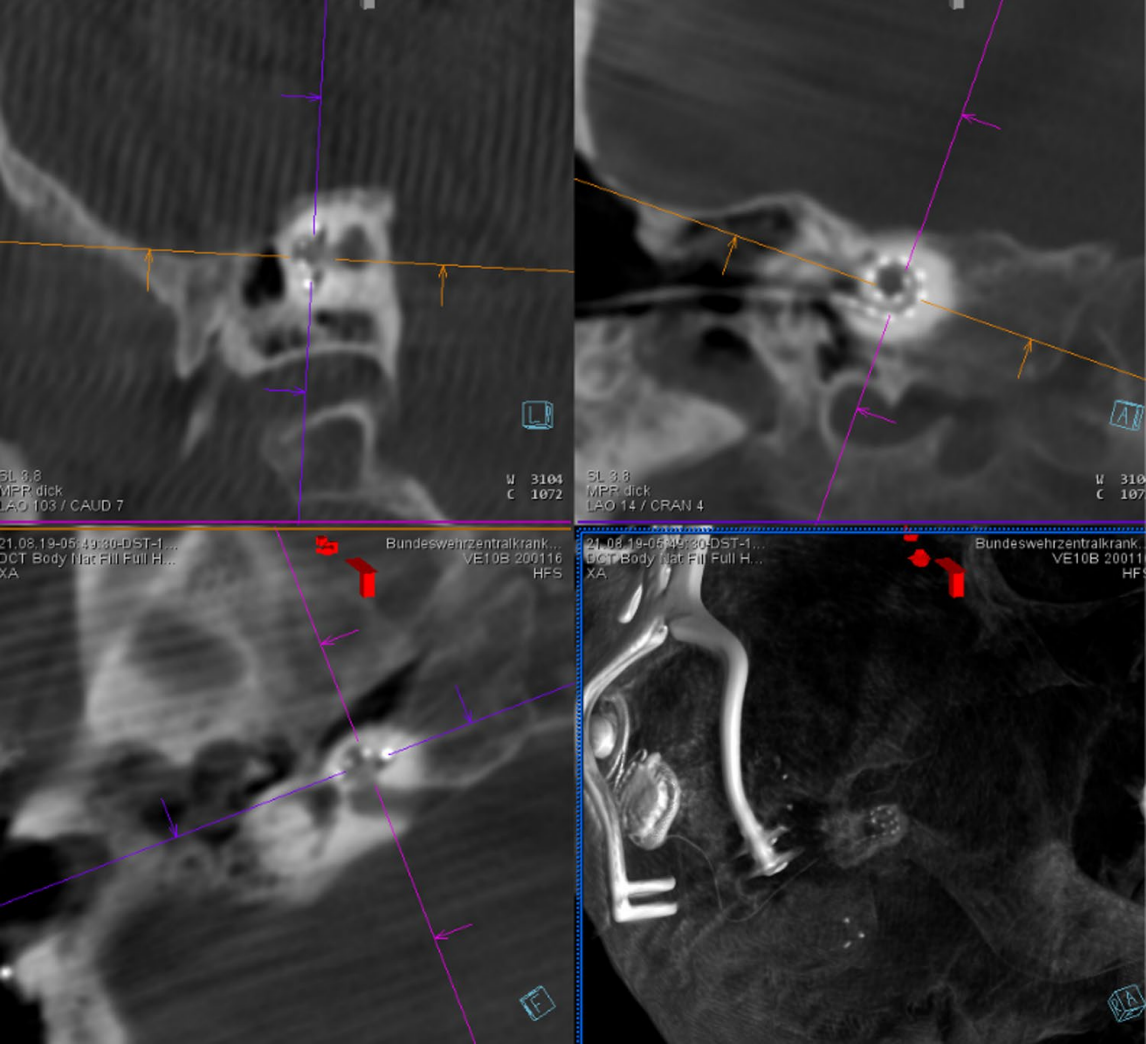
3D CT scan showing electrode position after insertion and before closure of the site

When these steps were completed, the team evaluated the images in the adjacent room and provided detailed information to the surgeon (Figs. 5, 6, 7, 8, 9, 10, 11).

The operation then continued.

If the operation is performed in small steps, this procedure must be repeated whenever necessary.

*Radiological findings in patient 4* Aplasia of the external auditory canal. The ossicular chain is poorly identifiable and malformed. There is evidence of discontinuity of the ossicular chain. Marked thinning of the lamellar bone cranial to the mesotympanum towards the neurocranium with unidentifiable areas of lamellar bone. The saccule are identifiable. Normal development of the cochlea with a length of 27 mm. The foramen ovale cannot be detected. Incomplete development of the semicircular canals. One semicircular canal with a saccular, can be identified. The internal auditory canal appears to be short and hypoplastic. Hypopneumatised mastoid cell system.

Details of the surgical procedure in patient 6.

## Results

### Safety

Cochlear implantation with an ARTIS Pheno in an interventional radiology suite is a safe procedure. Six out of seven ears have been successfully implanted. Based on current scientific knowledge and research, as well as patient findings, it was definitively not possible to implant one ear.

The objective of modern cochlear implant surgery is not only to place an implant, but also to place it correctly and completely. The insertion angle [1, 2] and especially the length of the electrode play an important role in personalizing cochlear implant surgery. A radiologist can provide useful advice, especially when it comes to determining the insertion angle.

In the cases presented here, the transfer of the surgical team and equipment from the operating theatre to the interventional suite three floors down in the same building did not pose any risk to the patients and did not affect their prognosis.

Before the patients woke up from anaesthesia, the electrode position had already been finally assessed and the surgeon and all other members of the surgical team had been informed of the successful placement. Once awake, the patients were also informed.

### Duration of surgery

An analysis of the duration of the various stages of cochlear implantation provided a variety of objective data, that are presented in Table 2. These results reflect a surgical procedure characterised by a series of interruptions and selective modifications, some of which require considerable (time) resources.

**Table 2:** .

	Additional time required (minutes)	Variability (minutes)
Time required for preoperative data assessment	22 [1]	18–22
Transfer of surgical material from the main operating room to the interventional suite	50.7 [1]	40–60
Presence of surgeon in the operating room until first incision	26 [1]	10–42
Incision-to-closure time	54 [1]	40–68
Number of intraoperative interruptions as a result of interactions with CT image data	1.8 [1]	1–3
Overall duration of intraoperative interruptions as a result of interactions with CT image data	4 [1]	12–23
Transfer of surgical material back to the main operating room	32 [1]	16–40
Time required for completing a patient-specific cochlear implantation report	8 [1]	6–10

[1] Mean value

## Discussion

The procedure described here requires considerable time and personnel resources. It is not useful as a standard approach, but it may be helpful especially in difficult situations [3–5].

When it comes to assessing the duration of surgery, it is difficult to compare the approach presented here with other surgical procedures. Regardless of the experience and expertise of the operating surgeon, the duration of surgical procedures performed in a conventional operating room in patients with challenging medical conditions cannot be accurately estimated. The duration of the procedures described here includes the time required for fluoroscopy and therefore includes the time for postoperative fluoroscopy, which is always performed after surgery but is not normally included in the operating time. In one patient, the operation included the removal of two basal cell carcinomas on the patient’s chest and associated plastic surgery, all performed under sterile conditions. These aspects distort the incision-to-closure times shown in the table. The site was closed only after final assessment of the implant position. In addition, this approach has only been used in a few cases to date. This means that the surgical team including the anaesthetist, had not previously worked closely with the radiology team in this specific setting. Perioperative procedures can certainly be improved.

Prior to surgery, specialists from the radiology and ENT departments discussed the patient’s data (CT images of the petrous bone, medical history, audiograms, surgical safety checklist, etc.) and jointly decided whether or not to perform the surgery in an multidisciplinary manner.

A debriefing was carried out to assess the needs of- the medical staff and to optimise procedures where possible. A material list was drawn up to ensure that all items needed for the procedure were available in the interventional suite and that no forgotten items had to be retrieved from several floors away.

Of course, there are many more ways to improve processes [2, 6]. Learning curve effects are likely to lead to improvements through increasing familiarity with the surgical environment, the suite, equipment positioning, optimising the use of sterile drapes, and using fluoroscopy under sterile conditions. Such effects have been described in detail for a number of novel surgical systems [5, 7–10] and can also be expected for the procedure presented here.

Documentation of cochlear implantation (coding of diagnoses and procedures, completion of the operative report) requires considerable time and effort in the immediate postoperative period. This is even more the case when the presence of anatomical variations or deviations from normal surgical procedures require a non-standard detailed patient-specific report. Documentation covers all stages of the operation and includes the collection and storage of fluoroscopy data and imaging procedure codes. In addition, intraoperative images and videos can be obtained at any time using an operating microscope (Arriscope). This data can be added to the patient’s medical record improving the quality of postoperative documentation [11]. We also use text templates to describe surgical procedures, which can be useful for creating documents in cases such as those reported here. Of course, it remains the responsibility of the surgeon to write a patient-specific operative report.

This overview has some limitations, one of which is the small number of patients (*n* = 6) and implanted ears (*n* = 7). A direct evaluation of the clinical and surgical benefits is not available since this surgical technique has not been used before. Although experience in the management of patients with anatomical variations and pre-existing medical conditions has been described, the number of patients is relatively small, with the exception of patients with a mastoid cavity following multiple surgical procedures. Moreover, the patients are geographically dispersed.

Experience with the ARTIS Pheno has mainly been reported by other specialists such as cardiothoracic surgeons [12–18].

The system has the advantage that the operating table is particularly stable and can accommodate patients weighing up to 280 kg. In addition, it allows surgeons to use fluoroscopy for percutaneous localisation procedures and reduces scanning time by 15% [12]. In thoracic surgery, for example, a significant reduction in operating time required for lymph node dissection has been reported [13]. HEARO, a robot that allows the insertion of electrodes in a computer-assisted procedure, is another example of attempts to perform surgical procedures in anatomical regions with multiple important structures in a controlled and safe manner. Another is to reduce tissue trauma by gaining controlled access to the cochlea and by placing the electrode in an optimal position. To this end, the ARTIS Pheno has been used to evaluate the insertion process in human cadavers under pre and postoperative fluoroscopy [14]. In neurosurgery setting, the ARTIS Pheno has been used to identify correct screw and rod placement and to decrease surgical complications and operating time [15, 16]. In another study, adaptive patient imaging on a clinical robotic CBCT system was found to be feasible during cardiac procedures, providing higher quality images while reducing the number of projections acquired by 90% compared to conventional cardiac imaging protocols [17].

Surgical systems using conventional or radiologically controlled techniques are therefore only partially comparable. The equipment involved and the small number of patients make it difficult to transfer such specialized procedures into routine clinical practice. The success of such interventional procedures depends on the personal experience and expertise of the surgeon, the anaesthetist, and especially the radiologist. Cost-effectiveness is also an issue that need to be addressed. Cost analysis can play a key role in user acceptance and translation into clinical practice. The discussion surrounding the daVinci Surgical System in recent years has highlighted the importance of costs [19, 20]. Future studies should evaluate further ENT procedures of similar complexity (e.g. skull base surgery and middle ear surgery) to focus more on the benefits of controlled surgical procedures and to demonstrate the advantages of 3D imaging systems such as the ARTIS Pheno.

## Conclusions

Surgical procedures that are performed in an operating theatre that offers immediate fluoroscopy during surgery and/or at the end of surgery are associated with a higher quality of care for patients.

The use of the ARTIS Pheno has already shown that the system can assist in the controlled insertion of cochlear implants in anatomically challenging situations and has considerable potential for further use in the field of otolaryngology.

However, the surgical equipment and supplies (neuromonitoring, surgical microscope, instrument sets, etc.) required for the procedure must be moved to the interventional suite.

It is difficult to compare the time required for surgery in a conventional operating room with that required for surgery in an interventional suite.

The positive results of the patients presented here encourage the use of this approach when an ARTIS Pheno or a similar system is available and a good interdisciplinary team is in place.
